# Dataset for assessing the impact of first-life lithium-ion battery degradation on second-life performance

**DOI:** 10.1016/j.dib.2025.112208

**Published:** 2025-10-27

**Authors:** Sadia Tasnim Mowri, Anup Barai, Muhammad Sheikh, Sangamitra Mohorana, James Marco

**Affiliations:** a WMG, University of Warwick, Coventry CV4 7AL, UK; b Electrical Power and Renewable Energy, College of Engineering and Technology, University of Doha for science and technology, Qatar; c Department of Metallurgical and Materials Engineering, National Institute of Technology (NIT), Warangal, Telangana, India

**Keywords:** Second life, Lithium ion battery, Grading, SoH, Degradation mode

## Abstract

This article outlines the objective, experimental design, and methodology used to evaluate the impact of first-life degradation on the second-life performance of lithium-ion batteries. To achieve this, 18 new cylindrical cells were subject to continuous electrical cycling at various charge-discharge rates until they reached 80% State of Health (SoH). The ageing process was carried out at a constant temperature of 25 °C. Reference Performance Tests (RPTs) using 1C and C/10 charge-discharge cycles at 25 °C were conducted on new cells and repeated at each ageing stage to quantify the SoH based on the level of retained capacity.

In this experiment, ageing the cells to 80% SoH is considered their first-life degradation phase. Once the cells reached 80% SoH, they were further cycled under a similar duty cycle at 25 °C to simulate second-life usage. After each duty cycle, RPTs were again performed using the representative second-life charge-discharge parameters to monitor the evolution of SoH.

The collected data was analysed to identify the degradation modes (DMs) of the cells at various SoH levels during their first life. Second-life data was used to examine how cells with similar first-life SoH and degradation modes performed during their second life. This dataset serves as a valuable resource for predicting battery lifespan based on charge and discharge rates and for developing automated battery grading systems that utilise first-life SoH and DM information. A complete description of the experimental method is discussed in detail within the associated research article [1].

Specifications Table

**Table:** 

Subject	Lithium Ion Cell
Specific Subject Area	SoH and DM Identification of New and first-life-aged cells, SoH identification of second life-aged cells
Type of Data	Battery Cycler (.csv) MATLAB files (.mat) Scanning Electron Microscope (SEM) images (.tif) Energy Dispersive X-ray Spectroscopy (EDX) data (.doc)
How the data were acquired	Electrochemical testing (Ageing and Performance Test) The constant cycle ageing of the 18 commercial LMG 50 cells was performed to reduce the SoH of the cell from 100 % to circa 80 % using different cycling conditions. The constant cycle of ageing is referred to as first-life ageing. During the first life testing, several RPTs were performed until the cells reached 80 % SoH. SoH of the cells was calculated using Eq. (1) by utilising the retained capacity from the RPT data. After the first life, the second life ageing of these 18 cells was performed using a real-world representative duty cycle. The real-world duty cycle employed for second-life testing is defined as the full equivalent month (FEM). Details of the FEM are provided in 5.2. During second-life testing, RPT assessment was performed after every FEM. SoH of the cells was calculated using Eq. (1) and utilising the capacity from the RPT data.
Data format	Raw
Description of Data Collection	Electrochemical data (e.g., capacity, voltage, temperature) was collected in .csv output file format. SEM data were collected from Aztec software.
Data Source location	Data source location Institution: Battery test laboratory, Energy Innovation Centre, WMG, University of Warwick. Coventry, United Kingdom, CV4 7AL
Data Accessibility	Repository name
Related Research Article	“Assessing The Impact of First-Life Lithium-Ion Battery Degradation on Second-Life Performance” https://doi.org/10.3390/en17020501

## 1 Value of Data

• This dataset comprises a comprehensive collection of ageing information for widely used, commercially available lithium-ion batteries. It includes regular capacity measurements until the cells' SoH declines from 100 % to circa 80 % during their first life and 80 % to circa 67 % during their second life. The dataset is well-suited for identifying the root cause of degradation i.e. degradation mode. Furthermore, it is appropriate for estimating the degradation rate of cells during first and second-life testing. Moreover, SEM images of the degraded cells might help researchers understand the degradation of the cell’s electrode.
• These datasets are applicable to a diverse group of researchers and engineers involved in long-term ageing tests, system design and battery modelling. The dataset could be valuable for researchers seeking constant cycling data or duty cycling data. Additionally, it is applicable to a wide range of researchers and engineers working on both first life and second-life ageing. These can be used for various purposes, such as predicting the degradation of lithium-ion batteries during the second life, developing battery degradation models, training artificial intelligence-based algorithms to estimate battery degradation in second life and automating battery grading techniques based on the cell's first life SoH and DM.

## 2 Objective

This dataset has been created to ascertain the influence of first-life DM on second-life performance and degradation. The objective of generating first-life test data was to create a group of cells at around 80 % SoH with the same DM; the second-life test was performed to identify the degradation behaviour of the cells with the same DM during second-life application.

## 3 Data Description

**Fig. 1:**
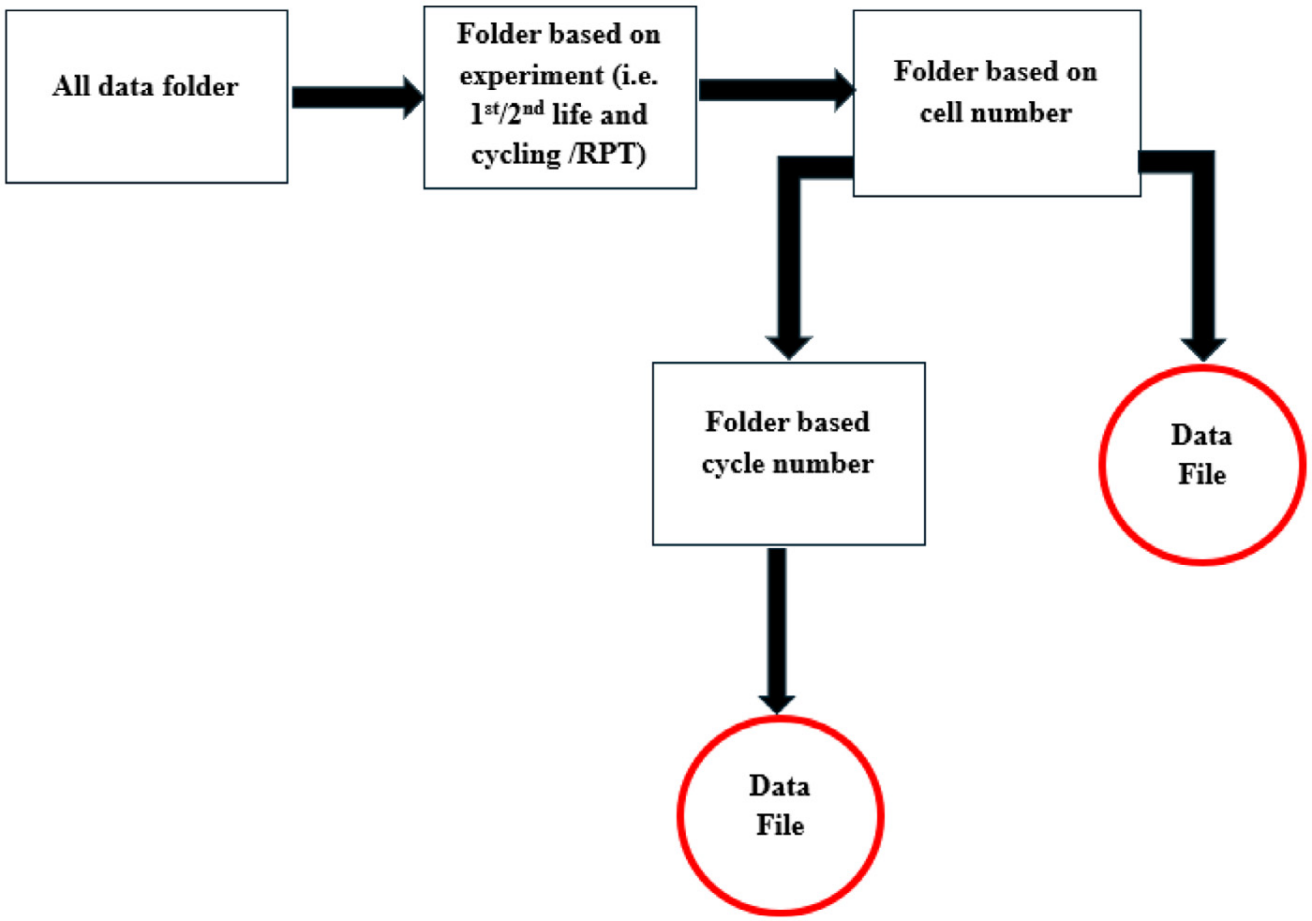
Graphical representation of the data location.

All the files are stored in the root folder named “Test_Data”. The folder contains the following sub-folders and files. A graphical representation of the data location is shown in Fig. 1.

1. 1st life cycling _Final
2. 1st life RPT _Final
3. 2nd life cycling _Final
4. 2nd life RPT_Final
5. Cross-sectional SEM and EDX
6. Surface SEM and EDX

### 3.1 1st life cycling data

**Fig. 2:**
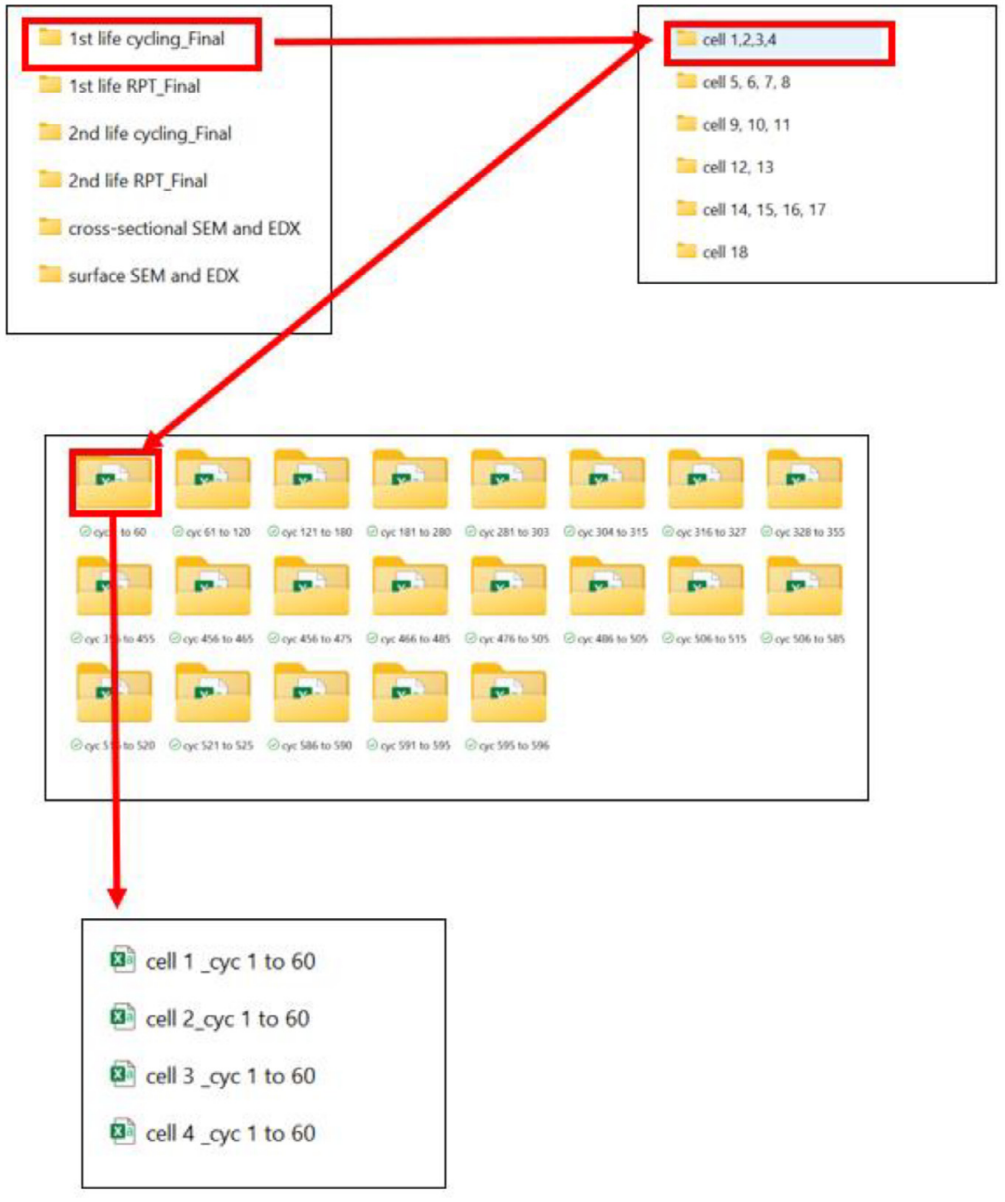
A demonstration to locate the 1st life cycling data for each cell.

This directory contains subfolders with cycling data for 18 cells, with each subfolder comprising CSV-formatted raw data. For example, the folder titled “Cell 1, 2, 3, 4” contains all first-life cycling data for Cells 1, 2, 3 and 4. Within each subfolder, data files for specific cycle numbers are organised. Every subfolder contains additional subfolders, which are denoted as “cyc 1 to 60,” “cyc 61 to 120,”and so on. The additional subfolder “cyc 1 to 60” contains .csv file with data from 1 cycle to 60 cycle. The folder structure is shown in Fig. 2.

### 3.2 1st life RPT data

This folder contains subfolders with the RPT data of eighteen cells. Each folder contains raw data in .csv format. Every .csv file in this folder has been converted to .mat file (MATLAB format). Each folder contains the cells denoted in the folder name. For example, folder name “Cell 1, 2, 3, 4” contains all the 1st life RPT data of the Cells 1, 2, 3, 4. The naming convention of these files follows the format:

Cellnn_RPT_0pAC_0pBC_100_0_Xcyc_25 degC
Cellnn: represents a unique identifier for each cell used in the experimental research, where nn related=s to the cell number
0pAC_0pBC: represents the charge-discharge rate i.e. 0.5C_0.3C, 0.7C_1C etc.
100_0: represents the state of charge
X cyc: The number of ageing cycles after which the energy capacity test was conducted
25 DegC: the ambient temperature of the cell, when the test was undertaken (for this data set, every RPT was done at 25DegC)

The structure of the raw data files is as follows:

Rows 1–15: header information of the test
Rows 16: Variable names
Row 17: Variable’s Unit
Row 18 - End: Test variable values

**Fig. 3:**
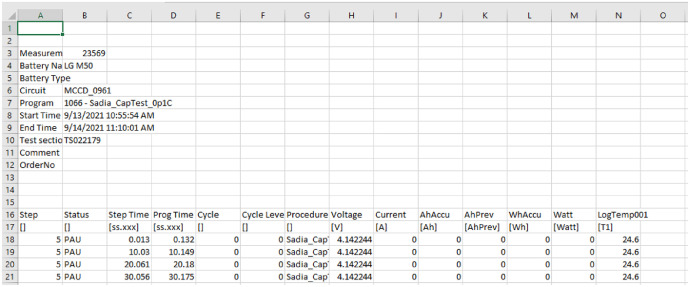
Definition of each column for Row 16.

The definition of each column for Row 16 with units is provided in Fig. 3.

Table 1 shows that Cell 1, 2, 3, 4 and Cell 9–11 were cycled at two different C-rate due to the slower degradation. Thus folder named Cell 1, 2, 3, 4 and Cell 9–11 contain RPT data after cycling with two different C-rates. For example, Cell 1, 2, 3, 4 were cycled with 0.5C_0.3C until 455cycles, after that Cell 1–3 were cycled with 0.5C_1C till 596 cycles and Cell 4 were cycled with 0.7C_1C till 525 cycles.

**Table 1:** First life data details.

Cell Number	1st C-rate	cycle data available with 1st C-rate	RPT data available after cycle no.	2nd C-rate	cycle data available with 1st C-rate	RPT data available after cycle no.
Cell 1	0.5C_0.3C	0–455	0, 60, 120, 180, 355, 455	0.5C_1C	456–596	475, 505, 585, 590, 595, 596
Cell 2	0.5C_0.3C	0–455	0, 60, 120, 180, 355, 455	0.5C_1C	456–596	475, 505, 585, 590, 595, 596
Cell 3	0.5C_0.3C	0–455	0, 60, 120, 180, 355, 455	0.5C_1C	456–596	475, 505, 585, 590, 595, 596
Cell 4	0.5C_0.3C	0–455	0, 60, 120, 180, 355, 455	0.7C_1C	456–525	505, 515, 520, 525
						
Cell 5	0.7C_0.3C	0–230	0, 13, 26, 39, 52, 65, 216, 220, 230			
Cell 6	0.7C_0.3C	0–247	0, 13, 26, 39, 52, 65, 216, 220, 230, 240, 245, 246, 247			
Cell 7	0.7C_0.3C	0–230	0, 13, 26, 39, 52, 65, 216, 220, 230			
Cell 8	0.7C_0.3C	0–230	0, 13, 26, 39, 52, 65, 216, 220, 230			
						
Cell 9	0.5C_2C	0–500	0, 75, 150, 225, 300, 500	0.7C_1.5C	501–570	520, 540, 570
Cell 10	0.5C_2C	0–500	0, 75, 150, 225, 300, 500	0.7C_1.5C	501–600	520, 570, 600
Cell 11	0.5C_2C	0–300	0, 75, 150, 225, 300	0.7C_1.5C	301–403	320, 370, 400, 403
						
Cell 12	0.7C_1C	0–200	0, 20, 80, 120, 140, 170, 190, 200, 201			
Cell 13	0.7C_1C	0–120	0, 20, 80, 120			
						
Cell 14	0.7C_0.5C	0–187	0, 20, 40, 80, 125, 175, 180, 185, 186, 187			
Cell 15	0.7C_0.5C	0–285	0, 20, 40, 80, 125, 175, 200, 230, 245, 255, 265, 285			
Cell 16	0.7C_0.5C	0–185	0, 20, 40, 80, 125, 175, 180, 185			
Cell 17	0.7C_0.5C	0–186	0, 20, 40, 80, 125, 175, 180, 182, 183			
						
Cell 18	0.7C_0.3C	0–280	0, 100, 180, 210, 230, 245, 255, 265, 280			

**Fig. 4:**
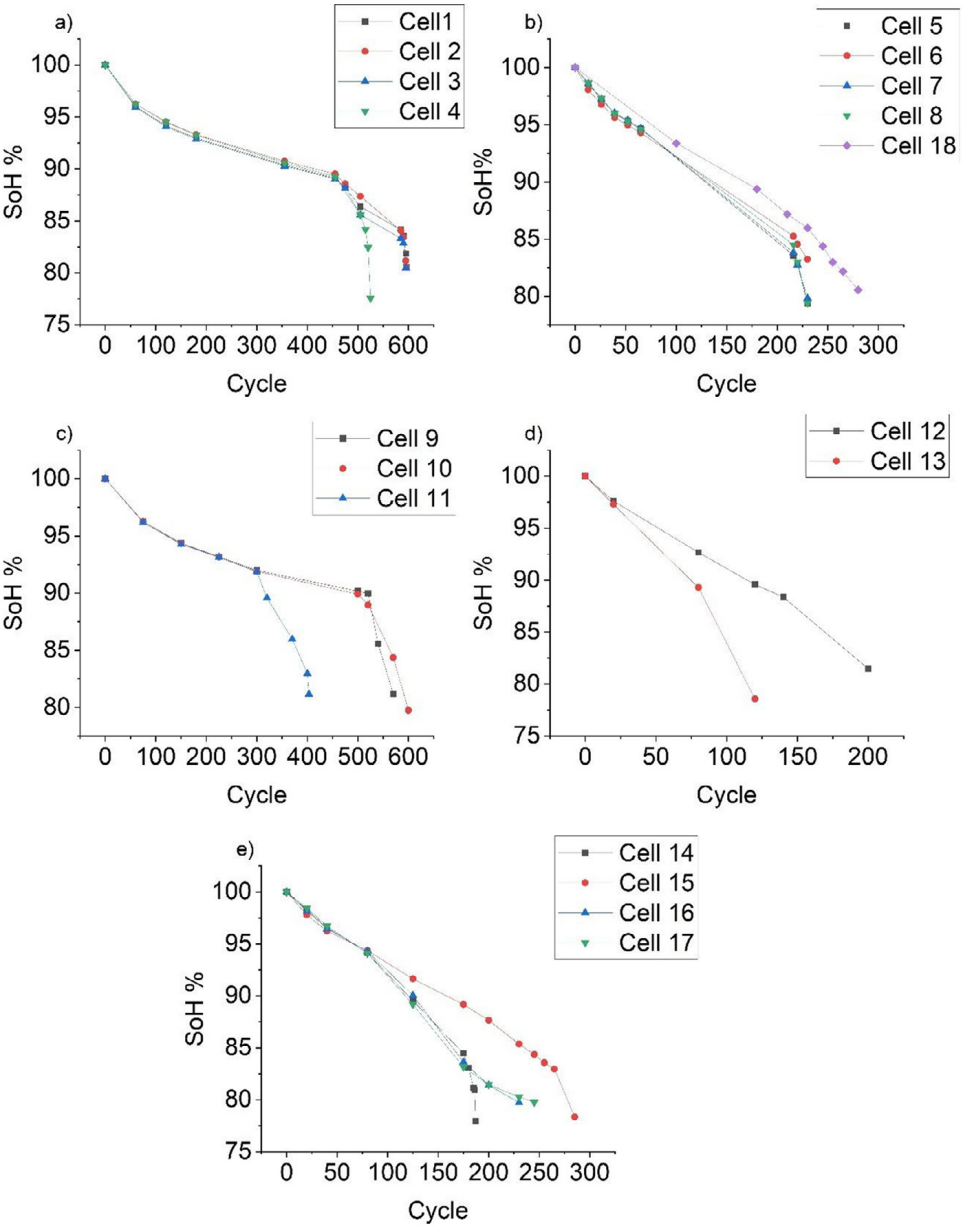
SoH of the cell provided in Table 1.

Initially, these two sets of cells were treated as separate groups and cycled under different load conditions. Specifically, cells 1–4 and 9–11 were tested with 0.5C_0.3C and 0.5C_2C C-rates. However, under these conditions, the cells degraded slowly, only reaching around 90 % SoH after 450–500 cycles. For cells 1–3, similar test conditions were used in their first life, i.e., they were cycled with 0.5C_0.3C and 0.5C_1C C-rates (Fig. 4(a)). These three cells required a similar number of cycles to reach the 80 % threshold.

At this stage of the experiment, the author was uncertain about the degradation rate of a cell cycled at 0.5C_1C after undergoing 0.5C_0.3C cycling. Considering the experimentation time an aggressive cycling condition was required to achieve 80 % SoH. Therefore, Cell 4 was subjected to a 0.7C_1C regime as the second C-rate to assess which condition would accelerate degradation more effectively. In addition, a reference group (Cells 1–3) had already been tested under similar cycling conditions. By applying different protocols, the author aimed to investigate how prior cycling conditions in the first life may influence degradation behaviour in the second life. SoH of the cells cycled with 0.7C_0.3C is shown in Fig. 4(b).

**Fig. 5:**
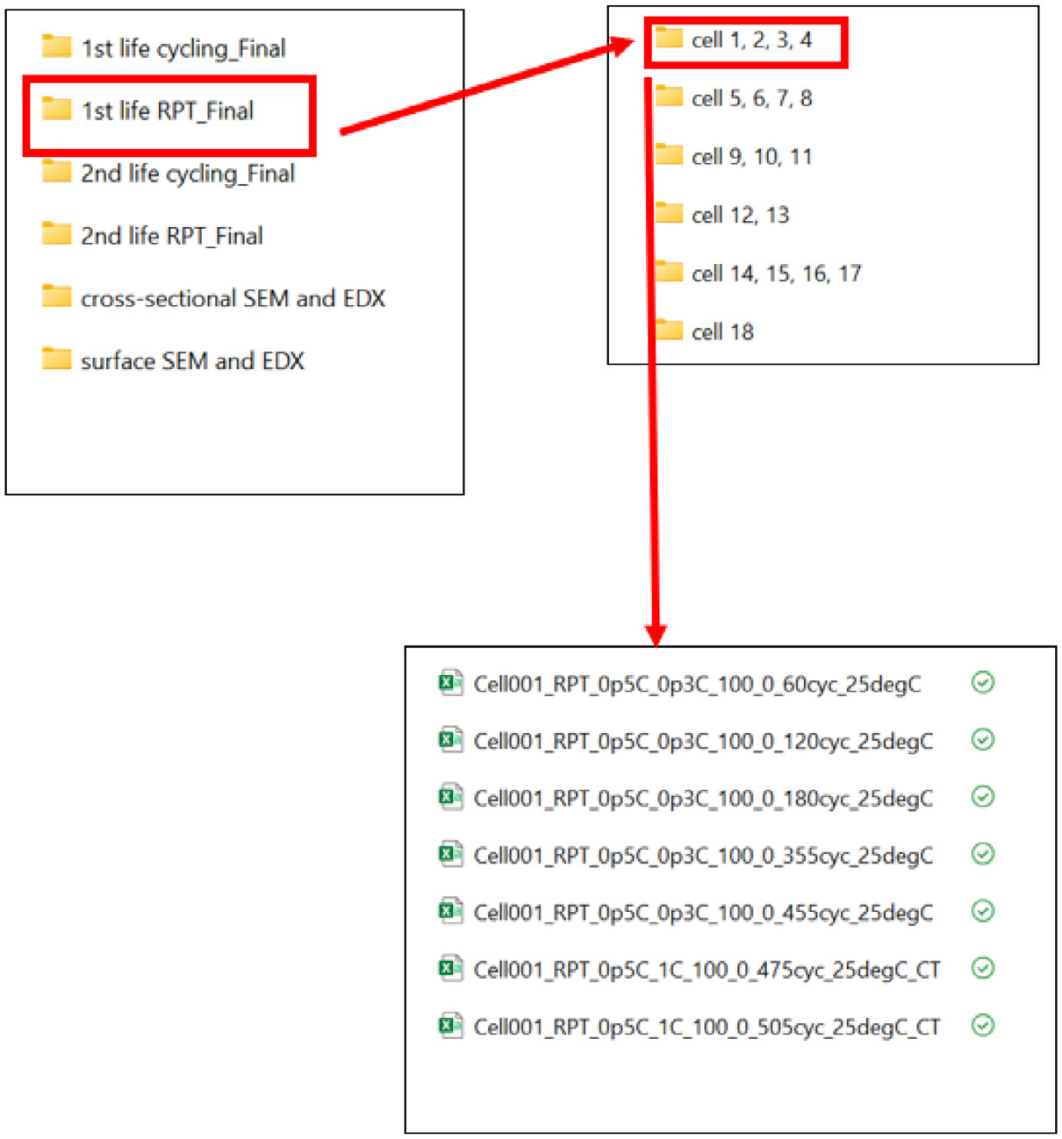
Demonstration to locate the 1st life RPT data for each cell.

Conversely, cells 9–11 were cycled under comparable conditions (0.5C_2C and 0.7C_1.5C C-rates). Despite being subjected to similar protocols, these cells exhibited different cycle counts to reach 80 % SoH. For example, Cell 9 reached 80 % SoH after 570 cycles, whereas Cell 10 required 600 cycles. Such variation is likely due to inherent cell-to-cell differences. In the case of Cell 11, a sharp decline in SoH was observed after 300 cycles (as shown in Fig. 4(c)), followed by channel failures that led to data loss for this cell. In contrast, Cells 9 and 10 continued cycling up to 500 cycles. These results highlight the discrepancies in cycle life observed among the three cells. Impact of to inherent cell-to-cell differences also observed for cells cycled with 0.7C_1C and 0.7C_0.5C as shown in Fig. 4(d) and (e). As shown in Fig. 1 location of the 1st life RPT data is shown in Fig. 5.

### 3.3 2nd life cycling data

**Fig. 6:**
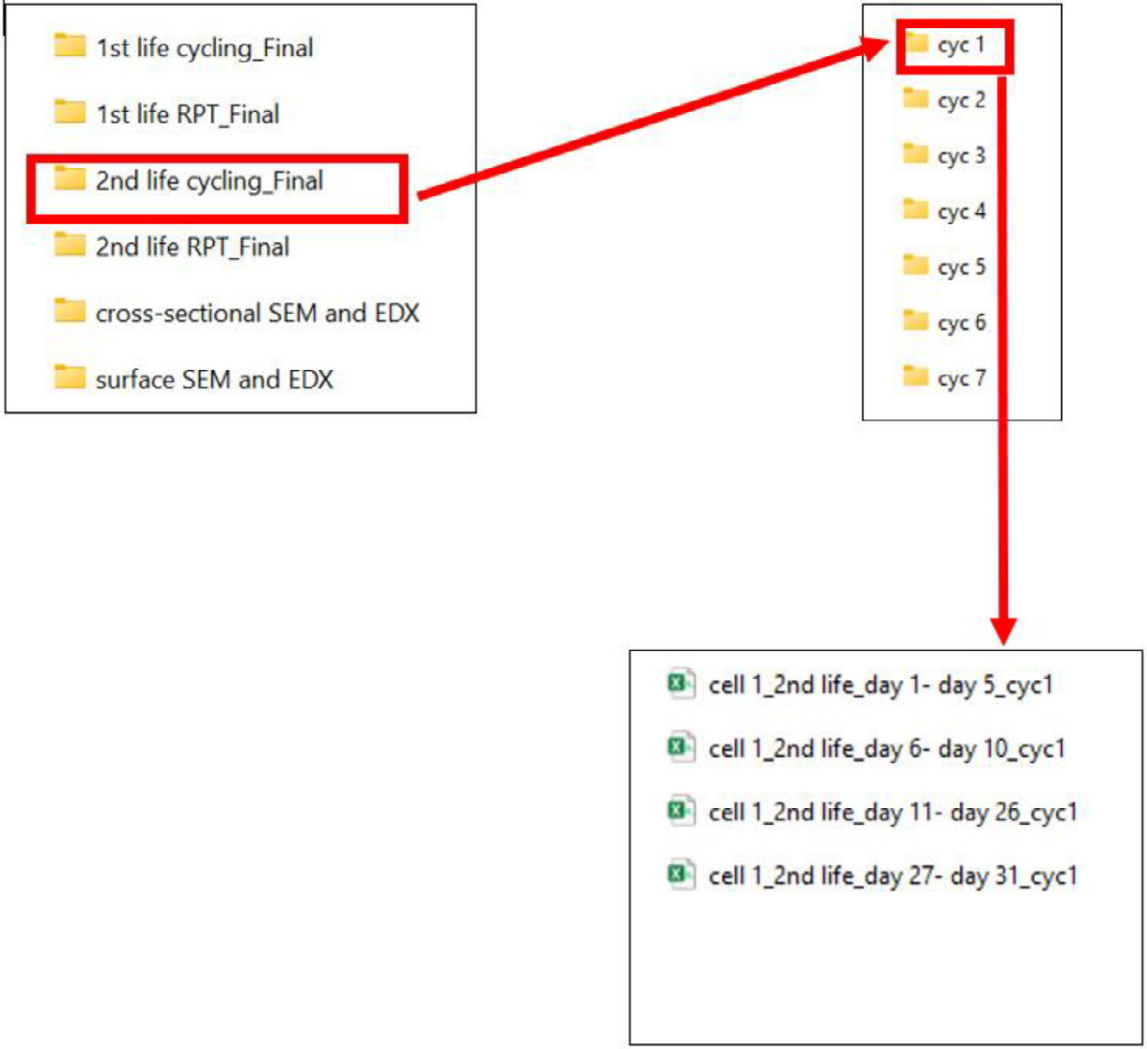
Demonstration to locate the 2nd life cycling data for each cell.

This folder contains subfolders with the second-life cycling data of 18 cells employed for second-life testing. Each folder contains raw data in .csv form. After completing cycle (cyc) 2 and RPT2, battery cycler failed; hence it was not possible to collect and analyse the cyc 2 and RPT 2 data. Though it was not possible to analyse the RPT 2 data, it is presumed (as similar profiles were added to all the cells) that any degradation resulting from the applied profile may become apparent in the subsequent RPT data. Data presented here as RPT 2 is RPT 3 data in real. To prevent reader confusion and present the data more coherently, the author decided to rename RPT 3 as RPT 2. Similarly, RPT 4,5,6,7,8 renamed as RPT 3,4,5,6,7, respectively. Further information is available in [1]. Fig. 1 indicates how to locate data files, while the first life RPT data corresponding to it is presented in Fig. 6. The naming convention of these files follows the format:

Cellnn_2nd life_dayX – dayY_cycM

Cellnn: represents a unique identifier for each cell used in the experimental research, where nn related to the cell number. cycM: represents the implementation of M number of full equivalent month (FEM) dayX – dayY: represents specific days of a FEM (each FEM consists of 31 days).

### 3.4 2nd life RPT data

**Fig. 7:**
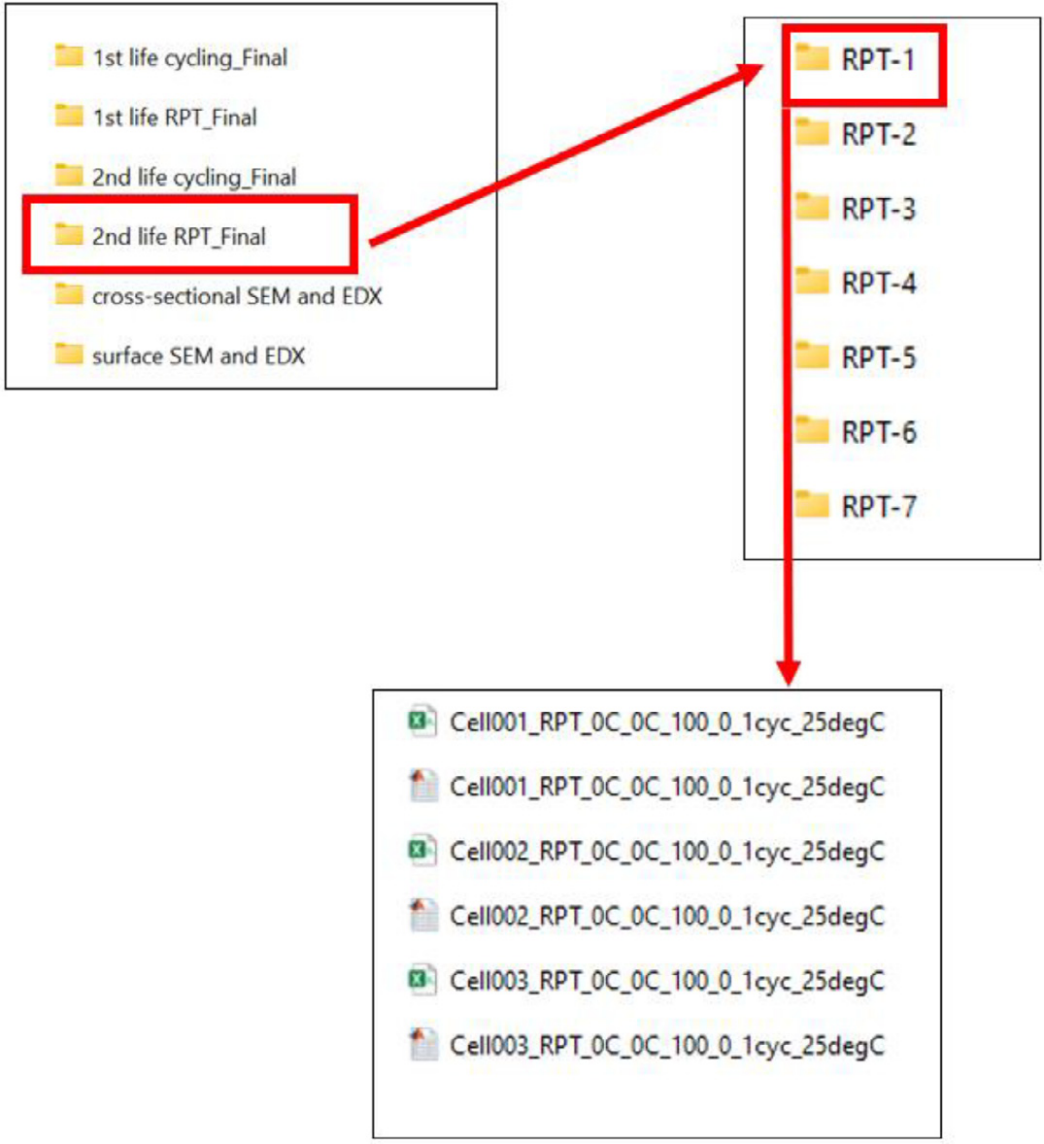
Demonstration to locate the 2nd life RPT data for each cell.

This folder contains subfolders with the RPT data of 18 cells. Each folder contains raw data in csv form, every csv file in this folder has been converted to a .mat file (MATLAB format). The folder defined as “RPT-n”: contains the RPT data after n^th^ full equivalent month (FEM). Details of the FEM is provided in 5.2. The location demonstration for the second life RPT data is presented in Fig. 7. List of second life RPT data is provided in Table 2.

**Table 2:** Second life data details.

Cell (a)	2nd life duty cycle for 1 Full Equivalent Month (FEM) (b)	RPT (c)
1–18	7 times	7 times

The naming convention of these files follows the format:

Cellnn_RPT_0C_0C_100_0_Mcyc_25degC
Cellnn: represents a unique identifier for each cell used in tshe experimental research, where nn related to the cell number
0C_0C: represents the duty cycle.
100_0: represents the state of charge
M cyc: The number of FEM after which the RPT was conducted
25 DegC: the ambient temperature of the cell, when the test was undertaken (for this data set, every RPT was done at 25DegC)

### 3.5 Cross-sectional scanning electron microscope (SEM) and EDX data

**Fig. 8:**
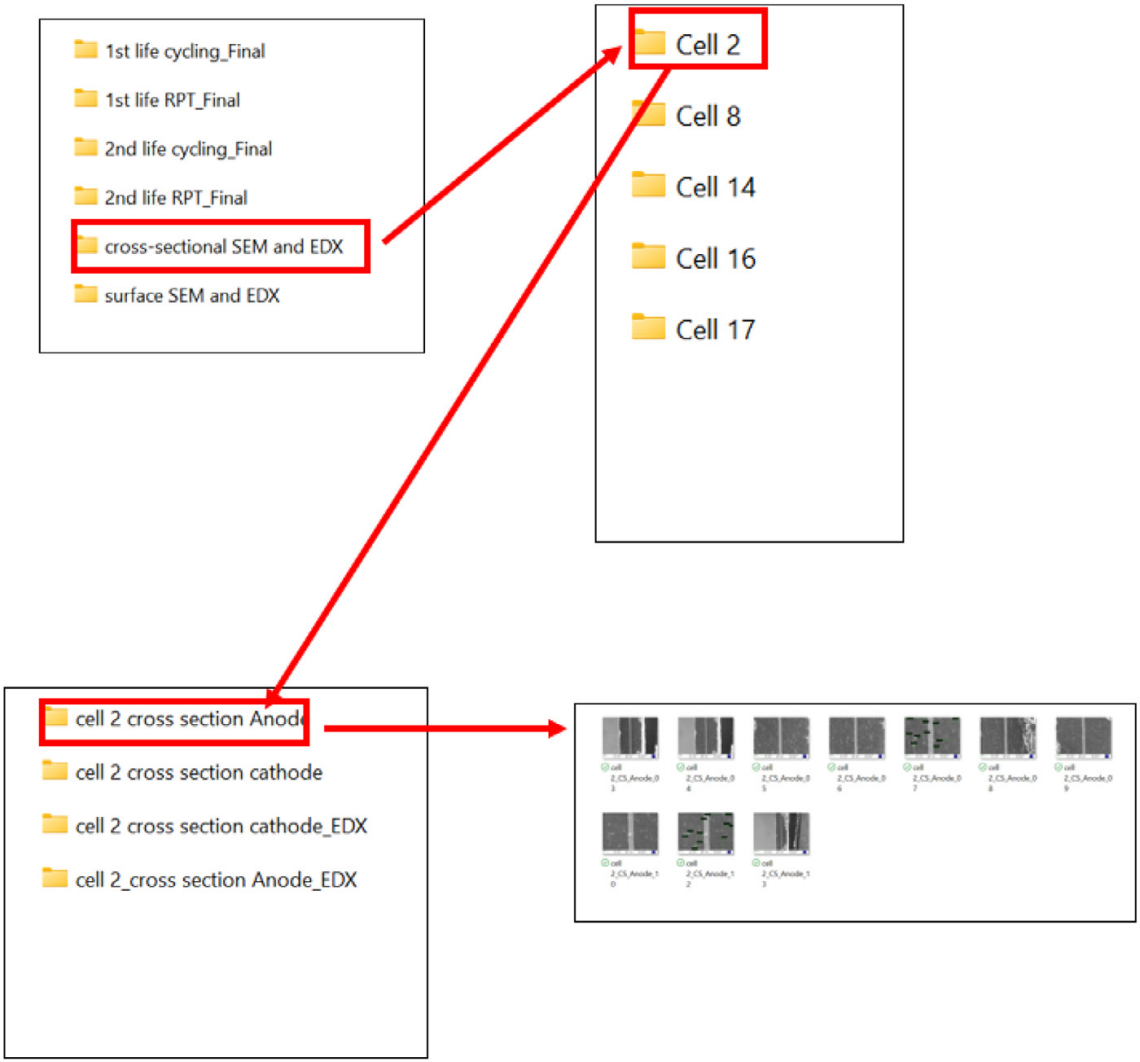
A demonstration to locate the Cross-Sectional SEM and EDX for cell.

Cross-sectional SEM electrode images of the cells that have completed their second life are available in the 'Cross-Sectional SEM and EDX' folder. This folder contains subfolders with the SEM and EDX data of each cell. Each folder contains subfolder named Cell nn cross section Anode, Cell nn_cross section Anode_EDX, Cell nn_Cathode, Cell nn_Cathode_EDX. Cellnn: represents a unique identifier for each cell used in the experimental research, where nn is related to the cell number. Folder named Cellnn cross sectional Anode, and Cell nn cross sectional Anode_EDX, contains SEM images and EDX data of the anode. Cell nn cross sectional Cathode, Cell nn cross sectional Cathode_EDX contains SEM images and EDX data of the cathode. Fig. 8 illustrates the location for cross-sectional SEM images and EDX data.

### 3.6 Surface scanning electron microscope (SEM) data

**Fig. 9:**
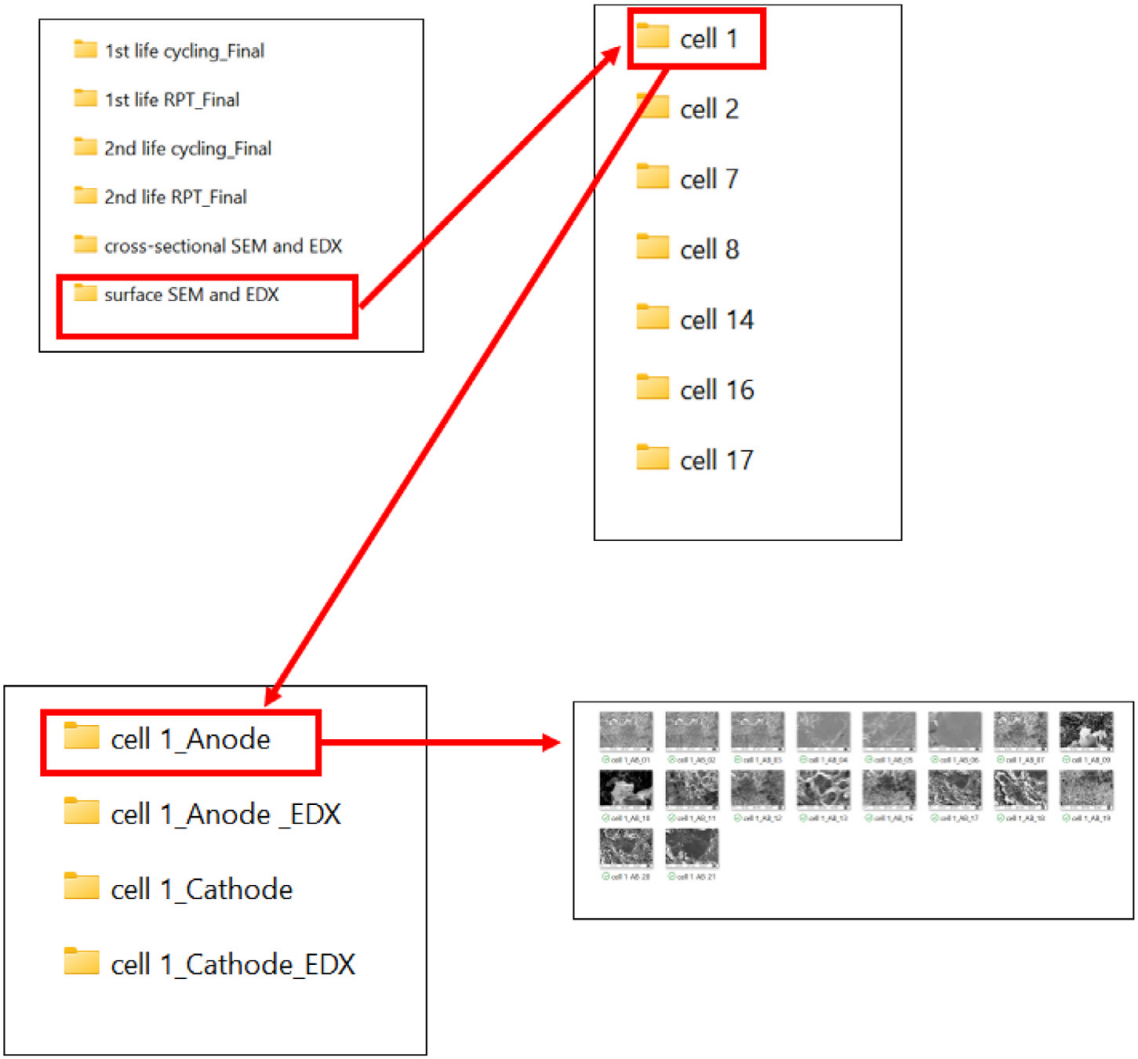
A demonstration to locate the Surface SEM and EDX for cell.

Surface SEM electrode images of the cells that have completed their second life are available in the 'SEM and EDX' folder. This folder contains subfolders with SEM and EDX data for each cell. Each subfolder includes further directories named *Cellnn_Anode, Cellnn_Anode_EDX, Cellnn_Cathode*, and *Cellnn_Cathode_EDX*, where *Cellnn* is a unique identifier for each cell used in the experimental research, with *nn* indicating the cell number. The *Cellnn_Anode* and *Cellnn_Anode_EDX* folders contain SEM images and EDX data for the anode, while the *Cellnn_Cathode* and *Cellnn_Cathode_EDX* folders contain SEM images and EDX data for the cathode. The location of the SEM images and correcponding EDX data is illustrated in Fig. 9.

## 4 Experimental Design, Materials and Methods

By 2030, the UK is expected to see a range of approximately 8 million to 11 million hybrid or electric vehicles (EVs) in use. By 2040, this figure has the potential to increase to as high as 25.5 million [2]. With the significant increase in the number of EVs, an increasing number of EV batteries will be retired from automotive use. As a result, the restoration and management of these retired batteries are becoming increasingly vital issues [3,4]. To address the issue, retired lithium-ion batteries are being deployed in second-life applications [4,5]. Batteries that have reached the end of their operational life cannot be directly employed for subsequent uses, such as a second-life application. This is primarily due to the irregular operating conditions and variable production processes, which can lead to several significant safety issues, including the risk of thermal runaway [6]. While sorting the cells, first life SoH is usually considered. However, the impact of the underlying mechanism that leads to degradation on the performance in a subsequent life remains undetermined [[6], [7], [8]]. Hence, the question remains as to whether obtaining more information beyond a fundamental SoH assessment is necessary before redeployment. However, a paucity of literature focuses explicitly on sorting retired cells based on their SoH and DM.

Moreover, limited experimental evidence exists regarding how retired cells age in the next life after regrouping based on both SoH and DM. This article presents a unique data set to identify the degradation behaviour of the cells with the same DM during the second life application. To address this question, a group of aged cells with a mixture of the same and different DMs is required. Different constant charge-discharge rates are employed to reduce the SoH of the cells from 100 % to 80 % SoH. RPTs were conducted until the cells reached approximately 80 % SoH (±1.5 %). SoH of the cells is measured employing Eq. (1). The nominal capacity (C_nominal_) of a battery is the capacity guaranteed by the manufacturer for a new cell operating under nominal conditions, such as nominal temperature (e.g., 25 °C), nominal discharge current e.g., one-hour discharge rate (1C), and being fully discharged from a fully charged state [9].

It is known that the energy capacity of a particular battery cell under different operating circumstances will vary. It is referred to as the actual capacity (C_actual_), which will vary at different operating and ageing states [9]. After the first life degradation, a second life degradation test was performed using a real-world duty cycle.

(1) $$SoH_{c}\;=\;C_{actual}/C_{nominal}$$

### 4.1 1st life constant cycling test

**Fig. 10:**
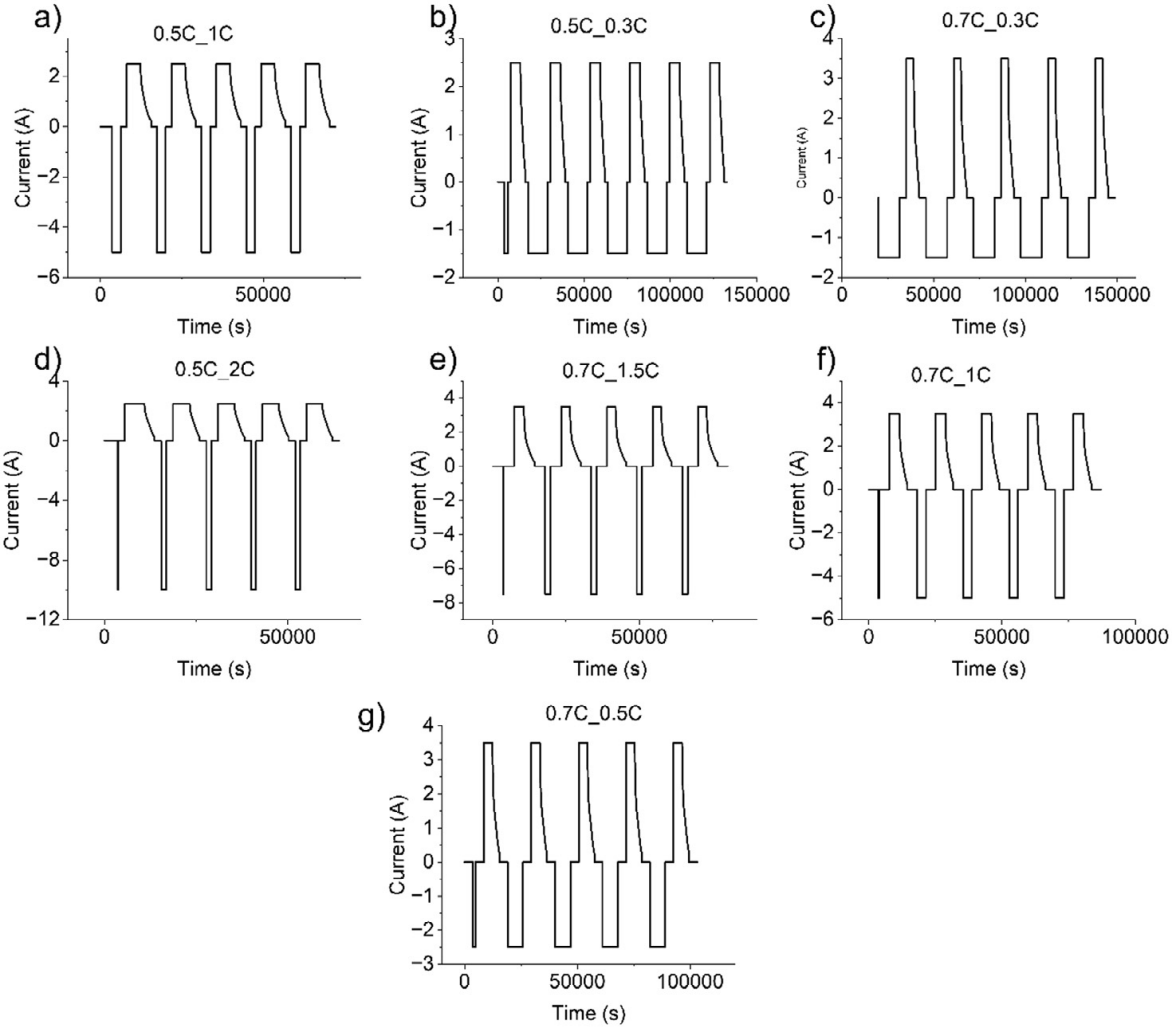
1st life Cycling profiles.

The cells underwent charging using the constant current-constant voltage (CC—CV) procedure for constant current cycling, followed by discharging using a constant current. The cycling temperature was maintained at 25 °C. During the charging phase, the cells were charged to 4.2 V using a constant current, and then, during the constant voltage (CV) step, the charging process was terminated when the current fell below C/20 (0.25A). Subsequently, after a one-hour resting period, the cells were discharged at their respective rates (e.g., 0.3C/0.5C/1C/1.5C) until the cell voltage reached 2.5 V. Following the discharge step, an additional one-hour rest period was introduced before resuming cycling. Representative cycling profiles used for 1st life cycling is shown in the Fig. 10.

### 4.2 2nd life degradation test

**Fig. 11:**
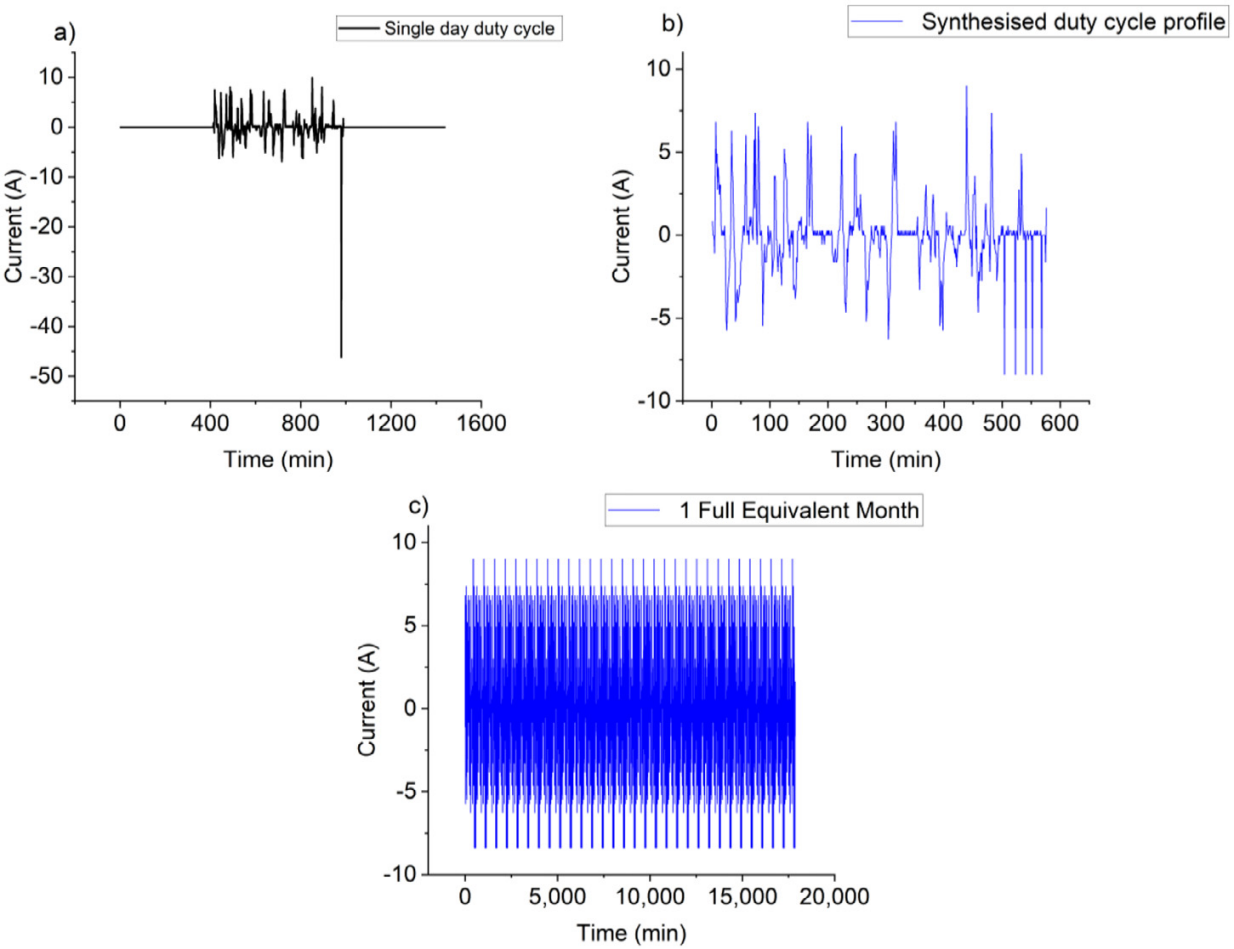
(a) Single-day duty cycle profile (b) Synthesised duty cycle profile (c) 1 FEM.

Throughout the second-life testing phase, RPTs were conducted following each FEM. The testing process continued until the cells reached an approximate SoH of 70 %. The second-life cycling procedures involved applying the profile illustrated in Fig. 11. This profile was derived from data collected from second-life battery packs engaged in grid services. Initially designed for a first-generation Nissan Leaf battery with a 33Ah capacity, the duty cycle profile needed scaling down to match the capacity level of the 21,700 NMC cells used in this study. The second life duty cycle was derived from second-life battery packs performing grid services. The original duty cycle profile is shown in Fig. 11 and was provided by an independent industrial partner. Among 720 provided cycles, the cycle shown in Fig. 11(a) was selected. The battery pack was a first-generation Nissan leaf battery with 33Ah capacity. Thus, the duty cycle profile needs to scale down to match the capacity level of 21,700 NMC cells used for this study. The profile (Fig. 11(a)) experienced an extended rest period, around 14 h, on that day. To accelerate the second life experiment, this rest period can be reduced. This is known to reduce the calendar life degradation and may affect the cycle life performance of the cell compared to the real-world [10]. However, this was deemed not to have a major implication for the research question being investigated as part of this research. As this study only investigates the degradation of cells in their second life that have undergone a similar second life profile, the influence of the reduced calendar life degradation is out of the scope of this article. After reducing the rest period, the duty cycle duration was reduced from 24 h to approximately 10 h, as shown in Fig. 11(b). Nissan Leaf battery cells have higher power capability than the cells used here. Therefore, on a couple of occasions (for Nissan leaf battery maximum 40A discharge current was observed (Fig. 11(a))), the high current pulse was scaled down to the maximum capability (approximately 9A discharge current (Fig. 11(b))) of the cell (LGM 50) used here to create a cycling profile representing one month of real-world cycling, the profile shown in Fig. 11(b) is repeated 31 times, as shown in Fig. 11(c). This synthesized monthly profile is considered as one FEM.

### 4.3 Reference performance test

The RPT of the cells was performed at 25 °C. RPT consist of a pseudo open circuit voltage (pOCV) test using C/10 current. Before the RPT, cells were charged first following CC—CV protocol. During the CC phase, the cell underwent charging at a rate of C/3 until the charge voltage reached its designated endpoint of 4.2 V. Following this, during the CV phase, the cell underwent charging at a voltage of 4.2 V until the current reached a value of C/20 (0.25 A). For the RPT; cells were discharged initially to 2.5 V, then charged to 4.2 V using C/10 current following the CC—CV protocol with a cutoff current C/20 (0.25A) at the CV phase. However, in the RPT data file reader might find a capacity test data at 1C charge and discharge along with the pulse power test data at 20 %, 50 % and 80 % SoC. This data were initially generated assuming might require for further degradation analysis. However, considering the volume of the work, data were not analysed further.

### 4.4 Cross-sectional SEM and EDX

Before collecting cross-sectional SEM images, samples were milled by using a broad-beam ion miller (Hitachi IM4000). To avoid moisture contact, samples were prepared in an argon-filled glove box before milling, and an airless transfer tool was used to transfer the sample from the glove box to the ion miller. Anodes were milled for 4 h with an ion beam current of approximately 120uA, discharge voltage of 1.5 kV and acceleration voltage of 6 kV. After that, SEM images of the cross-sectional samples were collected by a field-emission scanning electron microscope (FE-SEM) (Sigma, Zeiss), equipped with an energy-dispersive X-ray spectrometer (SEM-XmaxN 80, Oxford Instruments). For cross sectional image collection SE2 detector, 15 kV acceleration voltage and 30 µm aperture were employed.

### 4.5 Surface SEM and EDX

A FE-SEM (Sigma, Zeiss), equipped with an energy-dispersive X-ray spectrometer (SEM-XmaxN 80, Oxford Instruments), was also employed for surface SEM analysis. Certain substances exhibit rapid reactivity when exposed to air due to oxidation or moisture absorption from the surrounding environment [11]. For instance, Ni-rich NMC becomes susceptible upon contact with airborne moisture [12]. The surfaces of NMC particles can react with H_2_O and/or CO_2_, forming residual Li compounds such as Li_2_CO_3_ and LiOH [13]. These compounds result from interactions between NMC and ambient air. Brief exposure to air can change the Solid Electrolyte Interphase (SEI) composition in lithiated graphite [14]. To prevent moisture contact and preserve the microstructure, samples were prepared inside an argon-filled glovebox, maintaining oxygen and water concentrations below 0.1 ppm. Subsequently, the cycled electrodes were transferred to the SEM chamber using a specially designed airless transfer tool (Kammrath & Weiss). During image collection, an Inlense detector, a 5 kV acceleration voltage, and a 30 µm aperture were utilised. SEM analysis was conducted to assess morphological changes in the electrodes.

## Declaration of Competing Interest

There is no conflicts of interest.

## Figures and Tables

**Table:** 

Missing Data_1st life cycling		Missing Data_Second life cycling		Missing Data_Cross sectional
cell 11	cycling cyc 321 to 403	cell 1	cycling_2nd life_day 1–5_cyc2	Cell 17 _cross sectional EDX
cell 6	cycling cyc 246 to 247	cell 2	cycling_2nd life_day 1–5, Day 31_cyc2	
cell 12	cycling cyc 171 to 200	cell 3	cycling_2nd life_day 1–5,day 31_cyc2	
cell 14	cycling cyc 41 to 80	cell 4	cycling_2nd life_day 1–5, day 31_cyc2	
cell 15	cycling cyc 41 to 80	cell 5	cycling_2nd life_day 1–5,day 31_cyc2	
cell 16	cycling cyc 41 to 80	cell 6	cycling_2nd life_day 1–5,day 31_cyc2	
cell 17	cycling cyc 41 to 80	cell 7	cycling_2nd life_day 1–5,day 31_cyc2	
cell 14	cycling cyc 176 to 187	cell 8	cycling_2nd life_day 1–5,day 31_cyc2	
cell 15	cycling cyc 201 to 255	cell 13	cycling_2nd life_day 1–5,day 31_cyc2	
cell 16	cycling cyc 176 to 185	cell 14	cycling_2nd life_day 1–5,day 31_cyc2	
cell 17	cycling cyc 176 to 186	cell 16	cycling_2nd life_day 1–5,day 31_cyc2	
cell 18	cycling cyc 181 to 255	cell 17	cycling_2nd life_day 1–5,day 31_cyc2	
				
		cell 3	cycling_2nd life_day 31_cyc6	
		cell 4	cycling_2nd life_day 1_cyc6	
		cell 6	cycling_2nd life_day 1_cyc6	
		cell 7	cycling_2nd life_day 1_cyc6	
		cell 10	cycling_2nd life_day 1 to day 17_cyc6	
		cell 11	cycling_2nd life_day 31_cyc6	
		cell 13	cycling_2nd life_day 30 to 31_cyc6	
		cell 17	cycling_2nd life_day 31_cyc6	

## Data Availability

Mendeley DataTest_Data (Original data).

## Appendix

The testing phase of this research extended over a long period, during which the authors encountered issues such as cycler failure. Additionally, as the primary focus was on analysing RPT data to address the research questions, some cycling data from both the first-life and second-life testing phases were inadvertently not stored. To the best of the author's knowledge, the following data are missing.
